# Study on Cattle Trematodiasis and Related Risk Factors in Damot Sore District, Wolaita Zone, Southern Ethiopia

**DOI:** 10.1155/2023/6687665

**Published:** 2023-10-10

**Authors:** Isayas Asefa Kebede, Teshita Edaso Beriso, Tilaye Shibbiru Mengistu, Haben Fesseha Gebremeskel

**Affiliations:** ^1^School of Veterinary Medicine, Ambo University, Guder, Ethiopia; ^2^School of Veterinary Medicine, Wolaita Sodo University, P. Box: 138, Wolaita Sodo, Ethiopia

## Abstract

Trematodes are chronic, debilitating diseases in livestock, causing significant economic losses worldwide. From mid-December 2021 to May 2022, a cross-sectional study was carried out in the Damot Sore District to estimate the prevalence of trematode infections in cattle and associated risk factors. Trematode eggs were found in 100 of the 384 faecal samples tested, with an overall prevalence of 26.04% (95% CI: 21.88-30.69%). The prevalence for *Fasciola*, *Paramphistome*, and *Schistosoma* species were 12.50%, 5.21%, and 0, respectively. Moreover, the infection rate with two parasites was 8.33%. The data were then examined further using univariable and multivariable logistic regression analysis. As a result, age was the only potential predictor identified to influence trematode infections in cattle among the potential predictors considered. Furthermore, old cattle were more likely to be infected with trematodiasis nearly 12 times (OR = 11.5) that of young cattle, and this difference was statistically significant (*p* < 0.05), whereas other risk factors considered were statistically insignificant (*p* > 0.05). According to the findings of this study, cattle trematodiasis is a moderately common disease in the study area. As a result, additional research on the meteorological conditions of snail infection was forwarded, along with other points to reduce the disease problem in livestock production.

## 1. Introduction

Ethiopia has a hugely diverse terrain, a diverse variety of climatic topographies, and a plethora of agroecological zones that are appropriate for hosting a massive animal population [1]. It had the greatest livestock in Africa in 2020, with 65 million cattle, 40 million sheep, 51 million goats, 8 million camels, and 49 million poultry [2]. Despite having a huge livestock population, Ethiopia is unable to fully use these resources due to a multitude of reasons, including periodic droughts, infrastructural challenges, animal disease outbreaks, inadequate nutrition, poor husbandry practices, a shortage of trained manpower, and a lack of government policies for disease prevention and control [3].

In all parts of the world, parasitic infections have a significant influence on cattle production and welfare. Infections with the two types of internal parasites, liver flukes, and gastrointestinal nematodes are often considered the most harmful to cattle [4]. Helminthes, protozoa, and arthropod parasitic infections, in particular, have the potential to cause greater economic losses than bacteria and viruses, but the impact on animal owners is unknown [5].

Among parasitic diseases, trematode infections, primarily fasciolosis, are among the most economically significant helminth diseases affecting domestic ruminant production globally [6]. The category Digenea contains all parasitic trematode species in cattle [7]. Adult trematodes are frequently referred to as “flukes,” and the families that comprise prominent veterinary parasites include Fasciolidae, Dicrocoeliidae, Paramphistomatidae, and Schistosomatidae [8, 9].

The most significant flukes documented from various regions of the world are *Fasciola* (liver fluke), *Paramphistomes* (rumen/stomach fluke), and *Schistosoma* (blood fluke) [10]. Fasciolosis is one of the most prevalent parasitic diseases of domestic animals, affecting mostly cattle, sheep, goats, and, occasionally, humans. *Fasciola hepatica* (*F. hepatica*) and *Fasciola gigantica* (*F. gigantica*) are the two species most often implicated as etiological agents of fasciolosis. Only *F. hepatica* is a threat in Europe, the Americas, and Oceania, but the populations of both species overlap in many parts of Africa and Asia [11]. It is one of the most serious parasitic diseases affecting Ethiopian cattle production [12]. Fasciolosis is more common in young animals and is frequently chronic. Adult flukes in the bile ducts induce inflammation and cause bile duct blockage, liver tissue damage, and anemia. In this sense, both immature and adult flukes have a significant impact on the growth rate and feed conversion of young animals. There may be a decrease in milk output as well as a decrease in conception and pregnancy rates in cows [13].

Animal productivity, weight gain, and meat and milk output are all reduced by fasciolosis. Furthermore, chronic fasciolosis produces mild icterus, metabolic problems, and secondary infections due to weakened immunity, as well as liver condemnation during postmortem examination in slaughterhouses, whereas acute fasciolosis may result in fatalities [14].

Paramphistomosis is a pathogenic disease that affects domesticated ruminants and causes significant economic losses in the dairy and meat sectors. It is considered a neglected tropical disease, with the highest prevalence throughout tropical and subtropical regions, particularly in Africa, Asia, Europe, and Australia [15]. It has a wide geographical distribution, particularly in Thailand [16], Ethiopia [17], and Nigeria [18]. Different species of the Rumen fluke, *Paramphistomum*, predominate in different parts of the world. *Calicophoron calicophorum* is the most abundant species in Australia. *Paramphistomum cervi* is the most frequent species in countries as far apart as Pakistan and Mexico [19]. *Calicophoron daubneyi* is the most common rumen fluke in the Mediterranean and temperate areas of Algeria and Europe [20], and it has recently been identified as the most common rumen fluke in the British Isles [21, 22].

They are largely nonpathogenic, but clinical outbreaks have been reported to occur. Rumen flukes have a conical form that measures 5–12 mm by 2–4 mm; it has been observed that adults prefer the rumen and reticulum of ruminants, whereas immature parasites prefer the small intestine and abomasum [23].

*Schistosoma bovis*, *S. mattheei*, and *S. leiperi* can all cause schistosomiasis in cattle in Africa. In large ruminants, schistosomiasis is typically thought to be of minor relevance, and even when a high frequency of the parasite is found in slaughtered cattle, clinical indications of the disease are only observed rarely [8]. Infection, on the other hand, might cause serious clinical symptoms [7].

A thorough understanding of parasite epidemiology and interactions with hosts in a given environment and management system is required for a reasonable and long-term helminth control campaign [23, 24]. To reduce the economic losses caused by trematode infections, which are known as neglected tropical diseases and chronic debilitating diseases, various studies in various parts of the country have been presented thus far. Although considerable research has been conducted on various trematode infections in cattle and their prevalence has been reported for specific trematode parasites in Ethiopia, it is still necessary to generate periodic and current information on the concurrent prevalence of trematodes and the associated factors. Moreover, there is no previous study or documented information regarding snail-borne trematode infections in live cattle in the Damot Sore District, Wolaita Zone. As a result, the following objectives were set for the current study:

- Identifying and estimating the prevalence of major snail-borne trematode infections in cattle and assessing major risk factors that could contribute to snail-borne trematode infection in cattle in the study area

## 2. Material and Methods

### 2.1. Study Area

**Figure 1:**
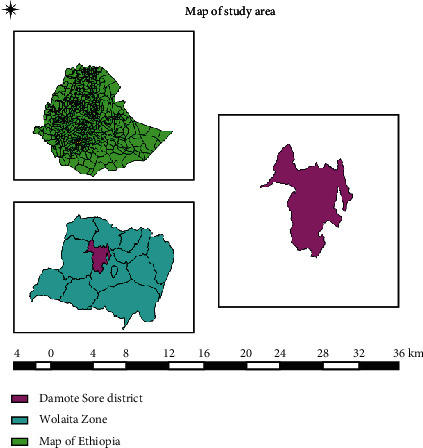
Location of the study area.

The study was conducted in the Damot Sore District from mid-December 2021 to May 2022, in the Wolaita zone, southern Ethiopia, 395 km southwest of the capital city of Ethiopia, Addis Ababa. Damot Sore District is bounded by Sodo Zuria in the southeast, Kindo Koysha in the west, Boloso Bombe in the northwest, and Boloso Sore district in the north (Figure 1). It is situated between 6° 51′ and 7° 35′ north latitudes and 37° 46′ and 38° 1′ longitudes, at an altitude of 1500 to 1800 meters above sea level. It receives 1200 millimeters of rain each year on average. The Damot Sore District has three different seasons: dry from November to February, light rains from March to June, and heavy rains from July to October [25, 26]. All year, the average annual maximum and lowest temperatures in the region are 21°C and 14°C, respectively. Mixed crop-livestock production is the most common agricultural method.

### 2.2. Study Population

The study population was cattle including both crossbreeds and indigenous breeds of all ages (older than 6 months) and both sexes. Before collecting samples, primary data on the breed, sex, body condition score, and age of the animals were recorded. Tooth eruption and wear, as described by Lahunta and Habel [27], were used to determine the age of the study animals. Accordingly, animals were divided into three groups based on their age: <2 years, 2–5 years, and >5 years. Animal body conditions were categorized as poor (score 1 to 3), medium (score 4 to 6), and good (greater than 6), based on observation of anatomical parts such as the vertebral column, ribs, and spines [28].

### 2.3. Study Design

A cross-sectional study was conducted to estimate the occurrence of snail-borne trematode infections in the study area from mid-December 2021 to May 2022.

### 2.4. Sampling Method and Sample Size Determination

Both districts and *kebeles* were selected purposefully based on prevailing livestock populations, transportation accessibility, and disease ecology as determined by the district's administrative body. The study animal was selected by a simple random sampling technique. Because there was no previous coprological study on snail-borne trematode infections in cattle in the study area, a 50% estimated prevalence was considered, as well as a 95% confidence interval, and a 5% required absolute precision was used to calculate the sample size. The sample size was calculated according to Thrusfield [29] formula given below.

(1) $$\begin{array}{c} n=\frac{1.96^{2}\times P\exp\;\left(1-P\exp\right)}{d2}, \end{array}$$

where *n* is the required sample size, *P*exp is the expected prevalence (*p* = 50%). *d* is the desired absolute precision (5%). *Z* = 1.96 for a 95% confidence interval.

(2) $$\begin{array}{c} \frac{n=1.96^{2}\left(0.5\right)\left(1-0.5\right)}{{\left(0.05\right)}^{2}}=\frac{3.84\times 0.25=384}{0.0025}. \end{array}$$

Overall, 384 cattle were selected and examined.

### 2.5. Faecal Sample Collection and Examination

During the first recording of the animal information, faecal samples (approximately 10 g) were obtained directly from the rectum of the animals or immediately after defecation using sterile disposable plastic gloves and placed in a 10% formalin-filled universal bottle. Each sample was serially numbered and labeled with the date, origin, breed, deworming history, age, sex, and body condition of the animal. Next, the samples were placed in an icebox and transported to the Wolaita Sodo University veterinary parasitology laboratory for coprological examination. Then, the sample was immediately examined and/or maintained at 4°C until it was processed and checked. Accordingly, the sedimentation technique was used to detect and count the presence of trematode eggs by dilution of the faecal suspension and sedimentation of the eggs, which are heavier than most of the faecal particles. Briefly, two grams of faeces was added to 42 ml of water in a graduated cylinder. The contents were then mixed thoroughly using a glass rod and were poured through a tea strainer to remove large debris. The solution was then further passed through a sieve (mesh aperture 210 mm) into a conical flask, and water was run through the sieve to ensure no eggs remained attached to the sieve. The filtrate was then allowed to sediment for 3 min after which the supernatant was siphoned off taking care not to disturb the precipitated matters. The latter was stained with two drops of methylene blue, and the entire sediment was placed on a slide covered with a cover slip and viewed under a compound microscope (Labomed) [7, 30].

A drop of methylene blue solution (1%) was added to the sediment to differentiate between eggs of *Paramphistome* species and *Fasciola* species. *Paramphistome* species eggs were stained with methylene blue, and the granules were translucent, whereas *Fasciola* species eggs were yellowish in color [8]. The differential EPG was calculated by dividing the specific parasite egg count in a sample of 3 g by 3.

### 2.6. Data Management and Analysis

During the collection of faecal samples from research animals, all data were recorded in field notebooks using predesigned or preprinted formats and subsequently input into a computer using a Microsoft Excel spreadsheet. Before statistical analysis, the dataset was extensively inspected for errors and/or mistakes and appropriately coded. The STATA version 14 computer programs were used to analyze the data, which was imported from Microsoft Excel. Descriptive statistics such as percentages were used to label the prevalence of trematode infection. Logistic regression analyses were conducted using trematode infection as an outcome or dependent variable against each of the explanatory or independent variables of the hypothesized risk factors (deworming history, breed, sex, age, body condition, and season). The explanatory variables with a *p* value ≤ 0.25 in univariable analyses were selected for multiple logistic regression analyses. The final multiple logistic regression models were built using a backward elimination approach. The chi-square test or odds ratio was used to measure the association or strength between possible risk variables such as age, deworming history, sex, physical condition, breed, location, and the prevalence of trematode infection. The odds ratio (OR) and its 95% confidence interval (CI) for the variables associated with the outcome variables were calculated. At a 95% confidence level, a *p* value of 5% indicated the presence of a significant association.

### 2.7. Ethical Considerations

Ethical authorization for the study was attained from Wolaita Sodo University, with the Veterinary Medicine Laboratory conferring to institute guidelines. Within each sampled area, the local community leader (LCL chairman) and a veterinary practitioner were informed about the research using a written letter from the respective district, and they assisted in mobilizing residents. At six of the sites, cattle owners brought their animals to the working area, or researchers traveled from house to house to obtain representative samples from each village, and in all cases, owners offered consent and were present when their livestock was sampled.

## 3. Results

### 3.1. Overall Prevalence

In the present study, an overall prevalence of trematodes 26.04% (95% CI: 21.88-30.69%) was found in 100 of the 384 cattle that were investigated. In this study, trematode infection prevalence was found in 35.00% of the crossbreeds and 25.00% of the local breeds, respectively. Similar to this, it was discovered that older animals had a greater prevalence of trematode infection (60.00%), followed by adult animals (23.97%), and young animals (11.54%). During the study period, the risk factors such as the sex of cattle, season, history of deworming, and body condition were used to evaluate the parasite spread (Table 1).

### 3.2. Univariable Logistic Regression Analysis

The results of univariable logistic regression analyses of the association between trematode infections and various risk factors are presented in Table 2. In the current study, the univariable analysis showed that only age was statistically significant (*p* < 0.05). However, other factors, such as deworming history, body condition, sex, breed, and season, were statistically insignificant (*p* > 0.05) (Table 2).

### 3.3. Multivariable Logistic Regression Analysis

Those potential risk factors (age, breed, BSC, and season) with *p* values less than 0.25 were subjected to multivariable logistic regression, and using the backward elimination technique, the final model was developed. Thus, age was the only risk factor associated with the disease, and hence, it was statistically significant (*p* < 0.05). Furthermore, the results also showed that the old animals were nearly 12 times (OR = 11.5) more likely to be exposed to trematodes than the young animals (Table 3).

### 3.4. Species-Specific Prevalence of Trematode Infections

The prevalence of single and mixed infections is estimated in this study and shown in Table 4. The prevalence for *Fasciola*, *Paramphistome*, and *Schistosoma* species was 12.50%, 5.21%, and 0, respectively. Mixed infections with *Fasciola* and *Paramphistome* were recorded in 32 cattle (Table 4).

### 3.5. Faecal Egg Count

The eggs were counted, and the egg counts were classified semiquantitatively as “low” (1–2 eggs), “medium” (3–4 eggs), and “high” (>4 eggs) (Table 5). Furthermore, for *Fasciola* and *Paramphistomum* eggs, the results were also assessed quantitatively by dividing the number of fluke eggs counted by the faecal weight to calculate the eggs per gram of faeces (EPG) [31].

## 4. Discussion

This is the first study to estimate the prevalence of snail-borne trematode infections in live cattle in the study area. Trematode parasitism is one of the major issues affecting ruminant productivity globally [32], and they have been reported as the main parasitic problem for cattle and other ruminants in many parts of the world, including Africa [33]. Trematode infections are common in areas that are conducive to their intermediate snail hosts [34]. Understanding the epidemiology of trematode infection in cattle is critical for reducing the risk of infection, particularly by improving management to avoid exposure to intermediate hosts. In this study, local breeds of cattle were the most prevalent because they are the predominant breeds in extensive farming systems [35]. There were more female (54.95%) animals sampled than male (45.10%) animals because more female animals are usually kept by farmers for herd growth and milk production [36].

In the current study, based on a coprological study, cattle in the Damot Sore District were found to be infected with cattle trematodes with an overall prevalence of 26.04%. To compare the study to previous works in the field, there was a lack of information on concurrent trematode infections in cattle from the study area. However, a higher prevalence of cattle trematode infections in coprological studies of 61.06% [37] and 60.42% [38] has been reported in other parts of the north-west of Ethiopia and elsewhere in tropical countries: 64.6% in north-central Nigeria [39] and 33% in Tanzania [40]. The differences in prevalence might be due to the variations in sample sizes and ecological and climatic conditions between the study areas.

Moreover, the lower prevalence of cattle trematode infections, whose intermediate hosts are aquatic snails, observed in the present study could be explained by the fact that the study was conducted in areas with no major permanent water bodies, as opposed to some of the other studies, which were conducted near permanent water bodies and irrigation activities. One of the most important factors that influence the occurrence of trematodiasis in a certain area is the availability of suitable snail habitats [8].

The current study also showed the prevalence of single trematode infections in cattle. Accordingly, the highest prevalence was found for *Fasciola*, followed by *Paramphistomes*, whereas there was no recorded case of *Schistosoma* in studied animals. The differences in the prevalence of fluke infection might be attributed to the biology of the parasite and their intermediate hosts in the study area. Moreover, the intermediate hosts of *Schistosoma* need permanent nonflow water bodies, which is absent from the current study sites.

The current study estimated that there was zero prevalence of *Schistosoma*. Because the *Schistosoma* parasite's intermediate hosts were always dependent on the permanent water body to maintain the parasite's life cycle, additionally, the eggs of *Schistosoma* need the presence of water for development, and the temperature may affect cercariae shedding. Furthermore, in snails, *Schistosoma* takes a long time to develop, and hence, high rainfall is a good predisposing factor for the emergence of these parasites [41]. Epidemiological studies on cattle schistosomiasis point to the disease's endemicity, particularly in places with large permanent bodies of water and marshy pastures [42]. Moreover, this may be due to variations in climate leading to the drying of the natural habitats of the intermediate host, the snail, and the larval stages of the parasite, which may not reach infective stages and may decrease their population, leading to the absence or decrease in the prevalence of the disease in the area.

The most prevalent genus of trematodes identified in this study was *Fasciola* at a rate of 12.50% in cattle. This figure was approximately in line with a report from Hawassa at 11.5% [43] and from East Wollega Zone at 15.90% [44]. This might be associated with the existence of a favorable ecological condition for intermediate hosts of *Fasciola* in the study area.

The prevalence of cattle fasciolosis in the Damot Sore District was moderately low when compared to the study conducted and recorded in the coprological prevalence of the same zone: 16.6% [45], 23.7% [46], 19.4% [47], and 16.75% [48] and coprological prevalence reported elsewhere in Ethiopia; 54.2% in Eastern Shoa [49], 23.7% in Kellem Wollega zone [50], 50.79% in and around Inchini town [51], 41% in and around Woreta [52], and 33.42% in North Gondar [53]; as well as an abattoir study of the same zone: 20.24% [45], 26.8% [47], and 30% [48]; and an abattoir survey of other parts of Ethiopia 27.25% in Hossana [54], 24.4% in Haramaya [55], 39.95% in and around Bahir Dar [56], 21.9% in Nekemte [57], 53.48% in Jimma [58], 20.3% in Addis Ababa [59], and 32.3% in Adwa [60].

Similarly, a high prevalence of fasciolosis was reported elsewhere across the world. A coprological study found 19.25% [61] and 25.46% [62] reported from Pakistan and 66.14% from Bangladesh [63], and an abattoir survey found 21.8% in Nigeria [64] and 28.0% in Hadejia [65]. However, the current fasciolosis figures were higher than those reported in abattoir studies in the same zone, which was 4.9% [66], and elsewhere in tropical counties, 6.07% [67]. This disagreement might be attributed to differences in ecological and climate conditions, the limited accuracy of diagnostic techniques, the use of different diagnostic techniques, variations in sample size, the study area, and livestock management systems.

*Fasciola* prevalence has been reported to vary over the years, mainly due to variations in the amount and pattern of rainfall [68]. Additionally, optimal base temperatures between the levels of 10°C and 16°C are necessary for snail vectors of *Fasciola* spp. These thermal requirements are also necessary for the development of *Fasciola* within the intermediate host. The ideal moisture conditions for snail breeding and the development of larval stages within the snails are provided when rainfall exceeds transpiration and field saturation is attained. Such conditions are also essential for the development of fluke eggs, miracidiae searching for snails, and the dispersal of cercariae [8].

The current study found that paramphistomosis was the second-most common trematodiasis in cattle, with a rate of 5.21%. The rate of paramphistomosis was considerably in line with a report from Hawassa at 6.7% [43] and elsewhere in the world: in Germany at 5.5% [69] and in Malaysia at 5% [70]. The present *Paramphistome*s prevalence recorded was lower than the previous reports of coprological studies: in Holeta Agricultural Research Center Dairy Farm at 10.2% [54], in northwest Ethiopia at 45.83% [38] and abattoir study prevalence in Gondar at 51.82% [71], in Jimma at 57.52% [72], and in the eastern part of Turkey at 8.95% [73]. This difference might be due to variations in the livestock management system, the absence of swampy areas, the effect of deworming, and variations in the study period.

In the current study, the rate of concurrent trematodiasis (*Fasciola* and *Paramphistome*s spp.) in cattle in the study area was obtained at 8.33%. The occurrence of coinfections in this study was parallel to the studies in Bangladesh with 9.34% [74] and Malaysia with 11% [70], in which liver fluke and some species of stomach fluke may occur together in ruminants. Additionally, this could be attributed to the similarity of conducive environments for both parasites and intermediate hosts in the study area.

On the contrary, this prevalence rate was lower than the rates of trematodiasis reported in a coprology study: 56.6% in Tanzania [75], 37.24% in India [76], and elsewhere at 88.1% [77], as well as the abattoir survey: 45.70% in Pakistan [78], and 38.0% elsewhere in the world [79]. The differences in prevalence among various studies might be due to variations in the geoclimatic conditions of these areas of study. The present study revealed a lower prevalence that might be due to the awareness of cattle owners and the use of anthelmintics [53]. Moreover, this discrepancy may be due to the improvement of the concerned veterinarian's and private veterinary drug shops' interventions to minimize prevalence and, hence, contribute to the reduction of the parasite's infections in the cattle.

The infection rate was analyzed based on sex and showed that females (27.01%) were more infected than males (24.86%). This justifies the fact that cows are less resistant to infection with fasciolosis due to milk yield during lactation time; there may be a loss that predisposes them to the complication of their immunity; and also, most people traditionally feed their lactating cows by collecting grasses that are grown around rivers and marshy areas during the dry season, to attain a high milk yield from cows [80]. On the other hand, the results of the present study revealed that the sex of the animal had no significant effect (*p* > 0.05) on the occurrence of concurrent trematodiasis.

This signifies that sex has no impact on the infection rate and that both male and female animals were equally susceptible and exposed to the disease. This could be because both sexes are exposed to the same pasture lands and watering points, resulting in equal disease development. This finding agreed with another research report from Ethiopia [38, 44] and elsewhere in Bangladesh [74]. However, this result disagreed with the study of *Fasciola* and *Paramphistomum* species in Indonesia [81]. This might be attributed to the management system, with males getting longer exposure out the door when females are kept indoors at the beginning of lactation [82].

In this study, an attempt was also made to analyze the prevalence of concurrent trematode infections in cattle based on age, and there were extreme variations by age category. Infection rates were significantly higher in animals older than 5 years (60.00%), moderate in adults (23.97%), and lowest in young animals (11.54%). The older animals were nearly 12 (OR = 11.5) times more likely to be infected than the younger animals. Likewise, the adult animals were almost two (OR = 2.417) times more infected than the young.

This is because the disease occurrences were chronic, and the aged animals harbored the parasites for long periods without showing typical signs of disease intervention. Also, the reason for this could partly be that prolonged exposure to metacercariae-contaminated pastures most likely causes heavy infections in old animals compared to young animals. Furthermore, older animals were more likely to be kept under extensive grazing than younger groups. This finding was supported by other researchers [39, 83–85], who showed a statistically significant association between the age of the cattle and the prevalence of trematodiasis, which was higher in old cattle than in young cattle, reflecting their greater length of exposure to infection.

Moreover, the results of the current study indicated that there was a strong relationship between age categories (*x*^2^ = 30.277, *p* = 0.001). The high prevalence of old animals could be attributed to a long exposure time as well as management practices in which old animals were transported long distances to graze in swamps, valleys, and floodplain areas compared to young animals. In addition, when the cattle get older, their immunity against trematodes might decrease [45]. However, the current finding disagreed with Aragaw and Tilahun [37], which reported the occurrence of a higher infection rate in younger animals compared to their older counterparts and rationalized that older animals would develop acquired immunity that resulted in resistance.

The distribution of concurrent trematode occurrences by season revealed that concurrent trematode infections were more common in dry conditions (28.02%) than in semidry conditions (22.05%). Seasonal variations may be due to unequal sample distributions between seasons. Additionally, this might be due to grazing practices during the dry season, whereby cattle graze in marshy areas, valleys, and floodplains, thus exposing them to contaminated pastures. Furthermore, during dry seasons, animals were forced to graze and drink in communal pastures and ponds, which pose a high risk of cross-contamination and infection, as well as the accessibility of *Fasciola* and *Paramphistome* species' intermediate hosts.

Consequently, as animals congregate on the same common pasture land and ponds due to insecurity of feeding during dry seasons, the incidence of encysted metacercariae ingestion increases, resulting in seasonal dynamics in the occurrence of concurrent trematode infections in cattle. Furthermore, this might also be due to a decrease in the number of anthelmintic treatments during dry seasons compared to semidry seasons. Transmission studies on *Fasciola* spp. revealed that some free-floating metacercariae may be suspended in the water and consumed by the definitive hosts [39]. The optimal development of fluke eggs into miracidia occurs at the start of the wet season, and development within the snail is completed by the end of the rainy season. Therefore, the shedding of the cercariae coincides with the dry season, and more animals graze around streams and ponds at that time, thereby predisposing them to infections. In addition to that, herders migrate in search of water and grazing during the dry season, and thousands of cattle often converge on the few ponds that fail to dry up [24].

A higher prevalence was recorded in crossbred cattle (35%) than in local breeds (25%). This wide gap could partly be due to local breeds having acquired a high degree of immunity as a result of repeated natural exposure to the disease over a longer period. The main manifestation of immunity was the suppression of worm fecundity. It was also reported that local cattle that naturally acquire infections are capable of reducing egg production. Furthermore, there was a difference in natural or innate immunity between indigenous and crossbred cattle [85]. However, there was no significant difference (*p* > 0.05) between cattle fluke infections and breeds. These findings agree with Yeneneh et al. [38], which report no significant difference between trematode infections and breeds.

Correspondingly, the prevalence of snail-borne trematodes in cattle was found to be higher in poor-condition animals and lower in medium-condition animals. This could be because poorly fed animals were more likely than well-fed animals to develop fasciolosis and paramphistomiasis. Poor animal health also reduced cattle's resistance to infection challenges. Additionally, the prevalence of trematodiasis in cattle was found to be higher in dewormed animals and lower in nondewormed animals. This could be attributed to inappropriate timing of treatments, the use of inappropriate drugs, underdosing, or flukes developing resistance to the commonly used anthelmintics.

The present study also showed that the animals differed in the shading of parasite eggs. Accordingly, 12.5%, 7.81%, and 0.52% of low, moderate, and high *Fasciola* eggs were shaded, respectively. Similarly, 9.38% and 4.17% of low and moderate *Paramphistome*s eggs were shaded, respectively. Furthermore, *Fasciola* and *Paramphistome*s were shaded concurrently at 3.13%, 3.91%, and 1.30% of low, moderate, and high, respectively. This could be attributed to the biology of parasites, the immunity of hosts, their reactions to parasites, and treatment interventions with anthelmintics.

## 5. Limitations of the Study

The current study period covers only the dry and semidry seasons and does not include the light and heavy rainy seasons to compare the parasite distributions over the year. This is due to the short study period and manpower.

## 6. Conclusion and Recommendations

The present study demonstrated that trematode infections are moderately prevalent in the study area. This implies that the district was prone to health problems related to trematodiasis in cattle, which might subsequently reduce the economic output from cattle and, hence, remain a major problem that could hinder the growth of cattle production in this area. This study identified that the age of the animals was a risk factor for cattle trematodiasis. Female cattle had a higher prevalence of cattle trematodiasis than male cattle, during the dry season rather than the semidry season, and in older cattle (>5 years) rather than younger (2 years) cattle. This occurrence is closely associated with the major feed resources in the study area, which were almost natural pastures in the form of grazing land and which were seasonally waterlogged. In addition, the area lacked clean piped water for animals, consequently increasing the chance of exposure to trematode infection. Moreover, epidemiologically, the area was favorable for the development and multiplication of intermediate hosts. The majority of the animals examined in this study had a low to moderate number of parasite eggs. Consequently, there was a need to institute adequate control programs in the study area. Based on the above conclusions, the following recommendations were forwarded:

- The role of veterinarians in giving professional advice concerning awareness creation about the disease, strategic application of deworming, and preventing animals from grazing in water reservoir areas should be improved
- The coprological examination should be backed up by additional diagnostic methods including postmortem and immunological diagnosis due to the restricted accuracy of coprological testing
- The character of different risk factors and the type of intermediate hosts involved in the prevalence of trematode infections should be studied further.

## Figures and Tables

**Table 1:** Prevalence of trematodes with potential risk factors in cattle (*n* = 384).

Variables	Categories	No.	NPA	Prevalence (%)	95% CI
Sex	Male	173	43	24.86	18.95-31.88
	Female	211	57	27.01	21.43-33.44

Age	Young	52	6	11.54	5.22-23.59
	Adult	292	70	23.97	19.40-29.23
	Old	40	24	60.00	44.10-74.04

Breed	Cross	40	14	35.00	21.77-51.03
	Local	344	86	25.00	20.69-29.87

Deworming history	Dewormed	64	17	26.56	17.11-38.79
	Nondewormed	320	83	25.94	21.41-31.04

Body score condition	Good	82	24	29.27	20.38-40.08
	Medium	190	43	22.63	17.21-29.16
	Poor	112	33	29.46	21.72-38.61

Season	Semidry	127	28	22.05	15.6-30.15
	Dry	257	72	28.02	22.84-33.85

No. = number of animals examined; NPA = number of positive animals; CI = confidence interval.

**Table 2:** Univariable logistic regression output of risk factors and their odds of exposure (*n* = 384).

Variables	Categories	No.	NPA	*p* value	*χ*2	OR	95% CI
Deworming history	Nondewormed	320	83	0.917	0.011	1.033	0.562-1.898
	Dewormed	64	17			ref	

Body condition	Poor	112	33	0.976	2.272	1.009	0.540-1.887
	Medium	190	43	0.245		0.707	0.394-1.268
	Good	82	24			ref	

Age	Old	40	24	0.001^∗∗^	30.277	11.5	3.984-33.198
	Adult	292	70	0.052		2.417	0.991-5.899
	Young	52	6			ref	

Sex	Female	211	57	0.632	0.230	1.119	0.707-1.772
	Male	173	43			ref	

Breed	Cross	40	14	0.176	1.861	1.615	0.807-3.234
	Local	344	86			ref	

Season	Dry	257	72	0.211	1.572	1.376	0.835-2.269
	Semidry	127	28			ref	

No. = number of animals examined; NPA = number of positive animals; *χ*2 = chi-square; OR = odds ratio; CI = confidence interval; ref = reference cell. ^∗∗^*p* < 0.05.

**Table 3:** Multivariable logistic regression model output of risk factors and their odds of exposure associated with trematodes in cattle (*n* = 384).

Variables	Categories	No.	Prevalence (%)	*p* value	OR	95% CI
Age	Old	40	60.00	0.001^∗∗^	11.546	3.947-33.778
	Adult	292	23.97	0.055	2.417	0.991-5.899
	Young	52	11.53		ref	

OR = odds ratio; No. = number of animals examined; CI = confidence interval; ref = reference cell. ^∗∗^*p* < 0.05.

**Table 4:** Specific prevalence of trematodes in cattle in Damot Sore District (*n* = 384).

A genus of trematode discovered		No positive	%N	95% CI
Single	*Fasciola*	48	12.50	9.54-16.22
	*Paramphistomum*	20	5.21	3.38-7.95
	*Schistosoma*	0	0	ND
Mixed	*Fasciola* combined with *Paramphistomum*	32	8.33	5.94-11.56
Total		100	26.04	21.88-30.69

%N = prevalence; CI = confidence interval; ND = not defined.

**Table 5:** The frequency range of parasite infections based on EPG.

Parasites	Low EPG (%)	Mild EPG (%)	High EPG (%)
*Fasciola*	12.50	7.81	0.52
*Paramphistomes*	9.38	4.17	—
Both	3.13	3.906	1.302

## Data Availability

The analyzed data during this study will be provided on request from the corresponding author (teshitae2602@gmail.com).

## References

[1] Zhongming Z., Linong L., Xiaona Y., Wangqiang Z., Wei L. (2013). *WMO Workshop on Climate Monitoring including the Implementation of a Climate Watch System in RAI with focus on Eastern and Southern Africa*.

[2] CSA (2020). *Agricultural Sample Survey 2019/20 [2012 E.C.]. Volume II report on livestock and livestock characteristics (private peasant holdings)*.

[3] Bayou K., Geda T. (2018). Prevalence of bovine fasciolosis and its associated risk factors in Haranfama municipal abattoir, Girja District, South-Eastern Ethiopia. *SM Veterinary Medicine and Animal Science*.

[4] Kowalczyk S. J., Czopowicz M., Weber C. N. (2018). Herd-level seroprevalence of *Fasciola* hepatica and Ostertagia ostertagi infection in dairy cattle population in the central and northeastern Poland. *BMC Veterinary Research*.

[5] Shitaye J. E., Tsegaye W., Pavlik I. (2007). Bovine tuberculosis infection in animal and human populations in Ethiopia: a review. *Veterinary Medicine*.

[6] Mage C., Bourgne H., Toullieu J. M., Rondelaud D., Dreyfuss G. (2002). *Fasciola* hepatica and Paramphistomum daubneyi: changes in prevalences of natural infections in cattle and in Lymnaea truncatula from Central France over the past 12 years. *Veterinary Research*.

[7] Van Wyk J. A., Mayhew E. (2013). Morphological identification of parasitic nematode infective larvae of small ruminants and cattle: a practical lab guide. *Onderstepoort Journal of Veterinary Research*.

[8] Gm U., Armour J., Duncan J. L., Dunn A. M., Fw J. (2003). *Veterinary Parasitology 2nd Ed*.

[9] Mas-Coma S., Valero M. A., Bargues M. D. (2009). Chapter 2 *Fasciola*, Lymnaeids and human Fascioliasis, with a global overview on disease transmission, epidemiology, evolutionary genetics, molecular epidemiology and control. *Advances in Parasitology*.

[10] Dreyfuss G., Alarion N., Vignoles P., Rondelaud D. (2006). A retrospective study on the metacercarial production of *Fasciola* hepatica from experimentally infected Galba truncatula in Central France. *Parasitology Research*.

[11] Sultan K., Desoukey A. Y., Elsiefy M. A., Elbahy N. M. (2010). An abattoir study on the prevalence of some gastrointestinal helminths of sheep in Gharbia governorate, Egypt. *Global Veterinaria*.

[12] OIE (2016). *World Animal Health Information Database (WAHID)*.

[13] Kanyari P. W. N., Kagira J. M., Mhoma J. R. L. (2017). *Prevalence of endoparasites in cattle within urban and peri-urban areas of Lake Victoria Basin, Kenya with special reference to zoonotic potential*.

[14] Eman K., Sherif M. B., Reda S. F. (2016). Molecular characterization of *Fasciola* hepatica infecting cattle from Egypt based on mitochondrial and nuclear ribosomal DNA sequences. *The Journal of Parasitology*.

[15] Roy S., Lyndem L. M. (2019). An in vitro confirmation of the ethonopharmacological use of *Senna* plants as anthelmintic against rumen fluke *Paramphistomum gracile*. *BMC Veterinary Research*.

[16] Sripalwit P., Wongsawad C., Wongsawad P., Anuntalabhochai S. (2007). High annealing temperature-random amplified polymorphic DNA (HAT-RAPD) analysis of three *Paramphistome* flukes from Thailand. *Experimental Parasitology*.

[17] Sintayehu M., Mekonnen A. (2012). Prevalence and intensity of Paramphistomum in ruminants slaughtered at Debre Zeit industrial abattoir, Ethiopia. *Global Veterinaria*.

[18] Dube S., Aisien M. S. (2010). Descriptive studies on Paramphistomes of small domestic ruminants in Southern Nigeria. *Zimbabwe Journal of Science and Technology*.

[19] Rangel-Ruiz L. J., Albores-Brahms S. T., Gamboa-Aguilar J. (2003). Seasonal trends of *Paramphistomum cervi* in Tabasco, Mexico. *Veterinary Parasitology*.

[20] Ferreras M. C., González-Lanza C., Pérez V. (2014). *Calicophoron daubneyi* (Paramphistomidae) in slaughtered cattle in Castilla y Le on (Spain). *Veterinary Parasitology*.

[21] Gordon D. K., Roberts L. C., Lean N., Zadoks R. N., Sargison N. D., Skuce P. J. (2013). Identification of the rumen fluke, *Calicophoron daubneyi*, in GB livestock: possible implications for liver fluke diagnosis. *Veterinary Parasitology*.

[22] Zintl A., Garcia-Campos A., Trudgett A. (2014). Bovine *Paramphistome*s in Ireland. *Veterinary Parasitology*.

[23] Rojo-Vázquez F. A., Meana A., Valcárcel F., Martínez-Valladares M. (2012). Update on trematode infections in sheep. *Veterinary Parasitology*.

[24] Taylor M. A., Coop R. L., Wall R. L. (2007). *Vet Parasitol*.

[25] National Metrological Agency (2012). Hawasa branch directorate of South Nation’s Nationalities and Peoples Regional State, Ethiopia, Annual Report.

[26] Wolayta Zone Finance and Economy Development Office (2013). The 2013 annual report on the Wolayta zone rural woredas economy, Annual report.

[27] DeLahunta A., Habel R. E. (1986). *Applied Veterinary Anatomy*.

[28] Nicholson M. J., Butterworth M. H. (1986). *A guide to condition scoring of zebu cattle*.

[29] Thrusfield M. (2018). *Veterinary Epidemiology*.

[30] Palmer D. (2013). *Detection of Trematode Eggs and Eimeria Leuckarti-Sedimentation Method (FEST)–Faecal Samples [Internet]*.

[31] Alstedt U., Voigt K., Jäger M. C. (2022). Rumen and liver fluke infections in sheep and goats in northern and southern Germany. *Animals*.

[32] Vercruysse J., Claerebout E. (2001). Treatment vs non-treatment of helminth infections in cattle: defining the threshold. *Veterinary Parasitology*.

[33] Malone J. B., Yilma J. M. (1999). *Predicting outbreaks of fasciolosis: from Ollerenshaw to satellites*.

[34] Copeman D. B., Copland R. S. (2008). Importance and potential impact of liver fluke in cattle and buffalo. *ACIAR Monograph Series*.

[35] Blench R. (1999). *Traditional livestock breeds: geographical distribution and dynamics about the ecology of West Africa*.

[36] Majekodunmi A. O., Fajinmi A., Dongkum C., Shaw A. P., Welburn S. C. (2014). Pastoral livelihoods of the Fulani on the Jos plateau of Nigeria. *Pastoralism*.

[37] Aragaw K., Tilahun H. (2019). Coprological study of trematode infections and associated host risk factors in cattle during the dry season in and around Bahir Dar, northwest Ethiopia. *Veterinary and Animal Science*.

[38] Yeneneh A., Kebede H., Fentahun T., Chanie M. Prevalence of cattle fluke infection at Andassa livestock research center in the north-west of Ethiopia. *Veterinary research forum 2012 (vol. 3, no. 2, p. 85)*.

[39] Elelu N., Ambali A., Coles G. C., Eisler M. C. (2016). Cross-sectional study of *Fasciola* gigantica and other trematode infections of cattle in Edu Local Government Area, Kwara State, north-Central Nigeria. *Parasites & Vectors*.

[40] Nzalawahe J., Kassuku A. A., Stothard J. R., Coles G. C., Eisler M. C. (2014). Trematode infections in cattle in Arumeru District, Tanzania are associated with irrigation. *Parasites & Vectors*.

[41] Mandal C. S. (2012). *Veterinary Parasitology*.

[42] Amsalu G., Mekonnen Z., Erko B. (2015). A new focus of schistosomiasis mansoni in Hayk town, northeastern Ethiopia. *BMC Research Notes*.

[43] Mariam T., Mohamed A., Ibrahim N., Baye D. (2014). Prevalence of Fasciolosis and Paramphistomosis in dairy farm and household in Hawassa town. *European Journal of Biological Sciences*.

[44] Tilahun Z., Nemomsa D., Himanot H., Girma K. (2014). Study on prevalence of Bovine Fasciolosis at Nekemte Veterinary clinic, East Wolega Zone, Oromia, Ethiopia. *European Journal of Biological Sciences*.

[45] Zewde A., Bayu Y., Wondimu A. (2019). Prevalence of bovine fasciolosis and its economic loss due to liver condemnation at Wolaita Sodo Municipal Abattair, Ethiopia. *Veterinary Medicine International*.

[46] Alemu A. (2019). Prevalence of dairy cattle fasciolosis in and around Wolayta Sodo, Southern Ethiopia. *Journal of Dairy Research & Technology*.

[47] Alemu A., Belay A. (2015). Fasciolosis: prevalence, evaluation of flotation and simple sedimentation diagnostic techniques and monetary loss due to liver condemnation in cattle slaughtered at Wolaita Soddo municipal abattoir, Southern Ethiopia. *Food Science and Quality Management*.

[48] Nebyou M., Solomon M., Fanta D., Alemayehu R. (2015). Cross-sectional study on bovine fasciolosis: prevalence, coprological, abattoir survey and financial loss due to liver condemnation at Areka Municipal Abattoir, Southern Ethiopia. *Journal of Veterinary Medicine and Animal Health*.

[49] Mohammed C., Nigussie L., Dugasa J., Seid U. (2018). *Prevalence of bovine fasciolosis and its associated risk factors in Eastern Shoa, Kuyu District Central Ethiopia*.

[50] Kebede B., Lemessa A., Hailu S., Habtamu T., Adugna T., Getahun F. (2017). Cross-sectional survey of bovine fasciolosis in and around Seyo District of Kelem Wollega Zone, Oromia Region, Western Ethiopia. *Austin Journal of Veterinary Science & Animal Husbandry*.

[51] Abdi A., Zerihun A., Desta B., Fanta D. (2015). Prevalence of bovine fasciolosis in and around Inchini town, West Showa Zone, Adaa Bega Woreda, Centeral Ethiopia. *Journal of Veterinary Medicine and Animal Health*.

[52] Tsegaye B., Abebaw H., Girma S. (2012). Study on coprological prevalence of bovine fasciolosis in and around Woreta, Northwestern Ethiopia. *Journal of Veterinary Medicine and Animal Health*.

[53] Yilma J. M., Mesfin A. (2000). Dry season bovine fasciolosis in northwestern part of Ethiopia. *Revue de Médecine Vétérinaire*.

[54] Getahun T. K., Siyoum T., Yohannes A., Eshete M. (2017). Prevalence of gastrointestinal parasites in dry season on dairy cattle at Holeta agricultural research center dairy farm, Ethiopia. *Journal of Veterinary Medicine and Animal Health*.

[55] Yusuf M., Nuraddis I., Tafese W., Deneke Y. (2016). Prevalence of bovine fasciolosis in municipal abattoir of Haramaya, Ethiopia. *Food Science and Quality Management*.

[56] Aregay F., Bekele J., Ferede Y., Hailemelekot M. (2014). Study on the prevalence of bovine *fasciolosis* in and around Bahir Dar, Ethiopia. *Ethiopian Veterinary Journal*.

[57] Alula P., Addisu K., Amanuel W. (2013). Prevalence and economic significance of bovine fasciolosis in Nekemte municipal abattoir. *Journal of Veterinary Medicine and Animal Health*.

[58] Dechasa T., Anteneh W., Dechasa F. G. (2012). Prevalence, gross pathological lesions, and economic losses of bovine fasciolosis at Jimma municipal abattoir, Ethiopia. *Journal of Veterinary Medicine and Animal Health*.

[59] Aragaw K., Negus Y., Denbarga Y., Sheferaw D. (2012). Fasciolosis in slaughtered cattle in Addis Ababa abattoir, Ethiopia. *Global Veterinaria*.

[60] Bekele M., Getachew Y. (2010). Bovine fasciolosis. *Ethiopian Journal of Applied Science and Technology*.

[61] Fayyaz S., Shahbaz M., Baboo I., Nazir S., Shaukat M. (2021). Epidemiology and risk factor analysis of Fasciolosis in buffaloes in district Bagh, Azad Kashmir, Pakistan. *Journal of Bioresource Management*.

[62] Khan M. K., Sajid M. S., Khan M. N., Iqbal Z., Iqbal M. U. (2009). Bovine fasciolosis: prevalence, effects of treatment on productivity and cost benefit analysis in five districts of Punjab, Pakistan. *Research in veterinary Science*.

[63] Karim M. R., Mahmud M. S., Giasuddin M. (2015). Epidemiological study of bovine fasciolosis: prevalence and risk factor assessment at Shahjadpur Upazila of Bangladesh. *Immunology and Infectious Diseases*.

[64] Ardo M. B., Aliyara Y. H., Lawal H. (2013). Prevalence of bovine *fasciolosis* in major abattiors of Adamawa State, Nigeria. *Bayero Journal of Pure and Applied Sciences*.

[65] Abubakar S., Yunusa I., Ahmad M. K. (2017). Prevalence of fasciolosis among cattle slaughtered at Hadejia Abattoir. *Bayero Journal of Pure and Applied Sciences*.

[66] Abunna F., Asfaw L., Megersa B., Regassa A. (2010). Bovine fasciolosis: coprological, abattoir survey and its economic impact due to liver condemnation at Soddo municipal abattoir, southern Ethiopia. *Tropical Animal Health and Production*.

[67] Chaouadi M., Harhoura K., Aissi M., Zait H., Zenia S., Tazerouti F. (2019). A post-mortem study of bovine fasciolosis in the Mitidja (north center of Algeria): prevalence, risk factors, and comparison of diagnostic methods. *Tropical Animal Health and Production*.

[68] Mungube E. O., Bauni S. M., Tenhagen B. A., Wamae L. W., Nginyi J. M., Mugambi J. M. (2006). The prevalence and economic significance of *Fasciola* gigantica and Stilesia hepatica in slaughtered animals in the semi-arid coastal Kenya. *Tropical Animal Health and Production*.

[69] Forstmaier T., Knubben-Schweizer G., Strube C., Zablotski Y., Wenzel C. (2021). Rumen (Calicophoron/Paramphistomum spp.) and liver flukes (*Fasciola* hepatica) in cattle—prevalence, distribution, and impact of management factors in Germany. *Animals*.

[70] Khadijah S., Ariff Z., Nurlaili M. R. (2017). *Fasciola* and Paramphistomum infection in large ruminants. *International Journal of Agronomy and Agricultural Research*.

[71] Ayalew G., Tilahun A., Aylate A., Teshale A., Getachew A. (2016). A study on the prevalence of Paramphistomum in cattle slaughtered in Gondar Elfora abattoir, Ethiopia. *Journal of Veterinary Medicine and Animal Health*.

[72] Abebe F., Behablom M., Berhanu M. (2011). Major trematode infections of cattle slaughtered at Jimma municipality abattoir and the occurrence of the intermediate hosts in selected water bodies of the zone. *Journal of Animal and Veterinary Advances*.

[73] Ozdal N., Gul A., Ilhan F. A., Deger S. (2010). Prevalence of *Paramphistomum* infection in cattle and sheep in Van Province, Turkey. *Helminthologia*.

[74] Saha S. S., Bhowmik D. R., Chowdhury M. M. (2013). Prevalence of gastrointestinal helminths in buffaloes in Barisal district of Bangladesh. *Bangladesh Journal of Veterinary Medicine*.

[75] Swai E. S., Mtui P. F., Mbise A. N., Kaaya E., Sanka P., Loomu P. M. (2006). Prevalence of gastrointestinal parasite infections in Maasai cattle in Ngorongoro District, Tanzania. *Livestock Research for Rural Development*.

[76] Kuchai J. A., Hidayatullah T., Chishti M. Z., Farakhnaz R. (2012). Trematodiasis in cattle and buffaloes of Ladakh (J&K), India. *Journal of Comparative Clinical Pathology Research*.

[77] Roy B., Tandon V. (2010). Trematodiasis in North-East India: A Study on the Spectrum of Digenetic Trematodes among Pigs, Buffaloes, Cattle Goats, and Sheep. *Indian Journal of Animal Health*.

[78] Kakar M. N., Kakarsulemankhel J. K. (2008). Prevalence of endo (trematodes) and ectoparasites in cows and buffaloes of Quetta, Pakistan. *Pakistan Veterinary Journal*.

[79] Arias M., Lomba C., Dacal V. (2011). Prevalence of mixed trematode infections in an abattoir receiving cattle from northern Portugal and north-west Spain. *Veterinary Record*.

[80] Gracy J. S., Collins D., Huey R. (1999). *Meat Hygiene*.

[81] Hambal M., Ayuni R., Vanda H., Amiruddin A., Athaillah F. (2020). Occurrence of *Fasciola gigantica and Paramphistomum* spp infection in Aceh cattle. *E3S Web of Conferences*.

[82] Balock F. C., Arthur R. J. (1985). A survey of fascioliasis in beef cattle killed at abattoirs in southern Queensland. *Australian Veterinary Journal*.

[83] Waruiru R. M., Kyvsgaard N. C., Thamsborg S. M. (2000). The prevalence and intensity of helminth and coccidial infections in dairy cattle in Central Kenya. *Veterinary Research Communications*.

[84] Keyyu J. D., Monrad J., Kyvsgaard N. C., Kassuku A. A. (2005). Epidemiology of *Fasciola gigantica* and amphistomes in cattle on traditional, small-scale dairy, and large-scale dairy farms in the southern highlands of Tanzania. *Tropical Animal Health and Production*.

[85] Pfukenyi D. M., Mukaratirwa S., Willingham A. L., Monrad J. (2006). Epidemiological studies of *Fasciola gigantica* infections in cattle in the highveld and lowveld communal grazing areas of Zimbabwe. *Onderstepoort Journal of Veterinary Research*.

[86] Asefa D. I. (2022). *A Cross-Sectional Study on Prevalence of Bovine Trematodiasis and Associated Risk Factors in Damot Sore District, Wolaita Zone, Southern Ethiopia. Wolaita Zone, Southern Ethiopia*.

