# Corrigendum to “Study on Cattle Trematodiasis and Related Risk Factors in Damot Sore District, Wolaita Zone, Southern Ethiopia”

**DOI:** 10.1155/japr/9792165

**Published:** 2025-07-26

I.A. Kebede, T.E. Beriso, T.S. Mengistu, H.F. Gebremeskel, “Study on Cattle Trematodiasis and Related Risk Factors in Damot Sore District, Wolaita Zone, Southern Ethiopia,” *Journal of Parasitology Research*, 2023, https://doi.org/10.1155/2023/6687665

In the article, there is an error in the first line of the abstract:

“Trematodes are chronic, debilitating diseases in livestock, causing significant economic losses worldwide.”

should read as follows:

“Trematode infections are chronic, debilitating diseases in livestock, causing significant economic losses worldwide.”

We apologize for this error.

